# The Effects of Erector Spinae Plane Block in Terms of Postoperative Analgesia in Patients Undergoing Laparoscopic Cholecystectomy: A Meta-Analysis of Randomized Controlled Trials

**DOI:** 10.3390/jcm9092928

**Published:** 2020-09-10

**Authors:** Chang-Hoon Koo, Jin-Young Hwang, Hyun-Jung Shin, Jung-Hee Ryu

**Affiliations:** 1 Department of Anesthesiology & Pain Medicine, Seoul National University Bundang Hospital, Seongnam 13620, Korea; vollock9@gmail.com; 2 Department of Anesthesiology & Pain Medicine, SMG-SNU Boramae Medical Center, Seoul 07061, Korea; mistyblue15@naver.com; 3 Department of Anesthesiology & Pain Medicine, Seoul National University College of Medicine, Seoul 03080, Korea

**Keywords:** erector spinae plane block, opioid, postoperative pain

## Abstract

Ultrasound-guided erector spinae plane block (ESPB), a recent regional analgesic technique, has been used to manage acute pain after surgery. The aim of this meta-analysis is to identify the benefits of ESPB in patients undergoing laparoscopic cholecystectomy (LC). The authors searched PubMed, EMBASE, CENTRAL, CINAHL, and Web of Science to identify all randomized controlled trials (RCTs) evaluating the effects of ESPB on postoperative pain after LC. Primary outcome was defined as 24 h cumulative opioid consumption. Secondary outcomes were pain scores and the incidence of postoperative nausea and vomiting (PONV). We estimated mean differences (MD) and odds ratio (OR) using a random-effects model. A total of 8 RCTs, including 442 patients, were included in the final analysis. Postoperative opioid consumption was significantly lower in the ESPB group than in the control group (MD −4.72, 95% CI −6.00 to −3.44, *p* < 0.001). Compared with the control group, the ESPB group also showed significantly lower pain scores and incidence of PONV. A separate analysis of RCTs comparing ESPB with oblique subcostal transversus abdominis plane (OSTAP) block showed that the analgesic efficacy of ESPB was similar to that of OSTAP block. The results of this meta-analysis demonstrated that ESPB may provide effective postoperative analgesia in patients undergoing LC.

## 1. Introduction

Laparoscopic cholecystectomy (LC) is commonly performed with more than 500,000 cases per year to treat gallbladder disease [1]. LC produces significant postoperative pain despite being a minimally invasive technique [2]. The pain following LC consists of somatic and visceral components, and several modalities have been tried to decrease postoperative pain [3].

In recent years, the advent of ultrasound-guided interfascial plane blocks has been reported in the area of regional anesthesia and pain management. One of the novel techniques introduced in the literature is the erector spinae plane block (ESPB). It was first described in 2016 for the management of thoracic neuropathic pain, and has subsequently been used for acute pain control after surgery [4]. In this technique, a local anesthetic is injected into the fascial plane below the erector spinae muscles. Although the mechanism of the block is still unclear [5], this novel block has become popular with the increasing number of randomized clinical trials providing its effect.

In the past year, publications referring to ESPB have increased significantly. Subsequently, meta-analyses have been published, supporting the analgesic efficacy of ESPB [6,7]. However, previous meta-analyses included various types of surgery, ranging from laparoscopic procedures to cardiothoracic surgery. Since the severity of pain may be varied according to the type of surgery, the results of those studies should be interpreted with caution. Therefore, we designed and conducted a systematic review and meta-analysis of randomized controlled trials (RCTs) to identify the benefits of ESPB in patients undergoing LC. This meta-analysis also aimed to investigate the efficacy of ESPB compared with other regional blocks.

## 2. Materials and Methods

### 2.1. Protocol and Registration

The authors performed the systematic review and meta-analysis according to the Preferred Reporting Items for Systematic Reviews and Meta-Analyses (PRISMA) guideline [8]. The predefined protocol was registered in the International Prospective Register of Systematic Reviews (CRD42020162437).

### 2.2. Eligibility Criteria

All RCTs evaluating the effects of ESPB compared with no block or other regional blocks on postoperative pain after LC were included. There were no restrictions on publication year, language, and region. The authors excluded nonrandomized studies of intervention, case reports, letters to editors, review articles, and animal studies. The primary outcome was defined as cumulative opioid consumption at 24 hour postoperatively. The secondary outcomes included pain scores at 12 h and 24 h after surgery and the incidence of postoperative nausea and vomiting (PONV).

### 2.3. Sources and Search

Two authors (C.H.K. and J.Y.H.) independently conducted a literature search (PubMed, EMBASE, CENTRAL, CINAHL, and Web of Science) to identify all RCTs evaluating the analgesic efficacy of ESPB in patients undergoing LC. The search terms consisted of Medical Subject Headings terms and keywords, including “erector spinae plane”, “erector spinae plane block”, “ESB”, “ESP”, and “ESPB”. Each result was combined by the Boolean operator “AND” or “OR”. Detailed search terms for each database are shown in Table S1. The search was performed until July 2020.

### 2.4. Study Selection, Data Collection Process, and Data Items

Two authors (C.H.K. and J.Y.H.) independently read the titles and abstracts of the articles to remove obviously irrelevant studies. Subsequently, the full texts of the articles were retrieved and reviewed to include studies that met the aim of this study. Data from the final included articles were extracted and summarized in a spreadsheet by two independent authors (C.H.K. and J.Y.H.). The extracted data included first author, publication year, sample size, local anesthetics, target spine level, patient-controlled analgesia (PCA) consumption, pain scores, and the incidence of PONV. In addition, GetData Graph Digitizer 2.26 (http://www.getdata-graph-digitizer.com) was used to digitize and extract the data from the graph. Any discrepancy was settled by discussion with the corresponding authors (H.J.S. and J.H.R.).

### 2.5. Risk of Bias in Individual Studies

Two independent authors (C.H.K. and J.Y.H.) assessed the quality of the final included articles using the Cochrane Collaboration’s tool for assessing risk of bias for RCT [9], which consists of random sequence generation, allocation concealment, blinding of participants, blinding of outcome assessors, incomplete outcome data, selective reporting, and other bias. Each bias was graded as low, unclear, or high. The corresponding authors (H.J.S. and J.H.R.) were consulted to make a consensus for any disagreements.

### 2.6. Summary Measures and Synthesis of Results

This meta-analysis was conducted using R version 3.6.1 (R Foundation for Statistical Computing, Vienna, Austria) [10] with “meta” package [11]. For continuous variables, mean difference (MD) and 95% confidence intervals (CI) were calculated. If data were expressed as the median and range (minimum to maximum or interquartile range), the mean and standard deviation were estimated using Wan’s formula [12]. The missing standard deviation was imputed from similar RCTs using the same intervention according to the previous meta-analysis [13]. For dichotomous variables, odds ratio (OR) and 95% CI were calculated. A continuity correction of 0.5 was applied to zero total event RCTs, which means that no patients in both groups experienced the outcome event [14]. A random-effects model was employed due to the anticipated clinical between-study heterogeneity. In case the number of combined studies was lower than 10, the Hartung–Knapp–Sidik–Jonkman method was used in the random-effects analysis to minimize the error rate [15]. The results of the meta-analysis were presented by a forest plot. An I^2^ statistic estimated the degree of heterogeneity among the final included articles. It was interpreted as no (0–25%), low (25–50%), moderate (50–75%), or high (75–100%).

## 3. Results

### 3.1. Study Selection

**Figure 1:**
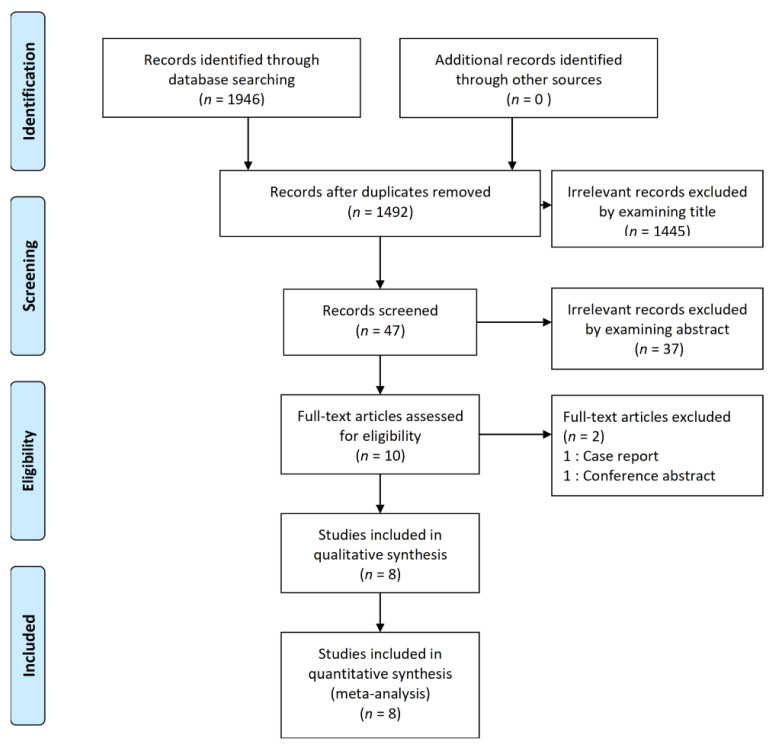
Flow diagram of included and excluded studies.

A total of 1946 articles were retrieved from the literature search, and 454 duplicate articles were removed. Subsequently, 1482 irrelevant articles were excluded after screening the titles (n = 1445) and abstracts (n = 37). Ten full-text articles were obtained and assessed, and 2 articles were excluded from the final analysis. The reason for the exclusion was as shown below: case report (n = 1), conference abstract (n = 1). Therefore, a total of 8 RCTs with 442 patients were included in the final analysis (Figure 1) [16,17,18,19,20,21,22,23] 199 patients were allocated to the ESPB group, 168 patients were allocated to the control group, and 75 patients were allocated to the oblique subcostal transversus abdominis plane (OSTAP) block group. Some studies have multiple groups [19,23]. Details of each RCT are summarized in Table 1.

### 3.2. Risk of Bias

**Figure 2:**
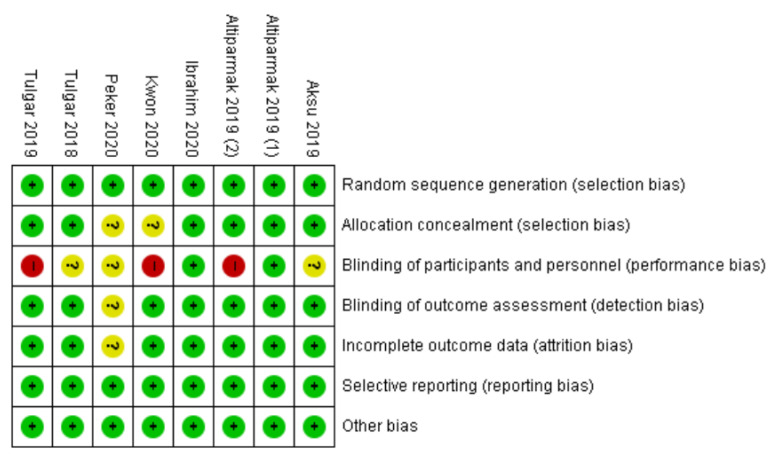
Risk of bias graph. Abbreviations: +, low risk of bias; ?, unclear risk of bias; −, high risk of bias.

The risk of bias is summarized in Figure 2, and the reasons for each judgement are described in Table S2. In all studies, patients were randomized to each group by specific methods. Allocation concealment was adequate in 6 out of 8 RCTs. The risk of performance bias was considered as “unclear” or “high” in 6 out of 8 RCTs. In those studies, patients in the control group received no sham injection, and thus these patients could recognize whether ESPB had been performed or not. In contrast, outcome assessors were blinded to the group in most RCTs. All RCTs were well controlled for attrition, reporting, and other bias.

### 3.3. Erector Spinae Plane Block vs. Control

**Figure 3:**
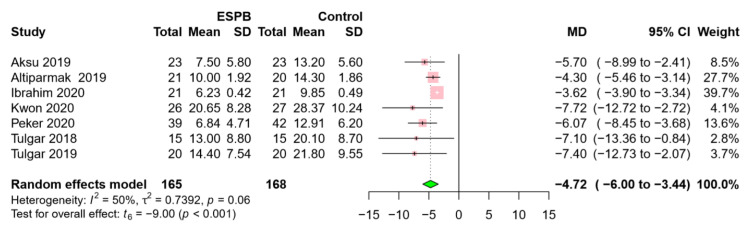
Forest plot for 24 h postoperative opioid consumption. Opioid consumption was significantly lower in the ESPB group than in the control group. Abbreviations: ESPB, erector spinae plane block; MD, mean difference; SD, standard deviation; CI, confidence interval.

Postoperative cumulative opioid consumption was reported in 7 RCTs, including 333 patients [16,17,19,20,21,22,23]. For postoperative analgesia, morphine was administered in 2 RCTs [16,19], tramadol was used in 4 RCTs [17,21,22,23], and fentanyl was used in 1 RCT [20]. The amounts of tramadol and fentanyl were converted to morphine-equivalent doses for data synthesis and analysis. For example, 100 mg intravenous tramadol or 100 mcg intravenous fentanyl was equivalent to 10 mg intravenous morphine [24,25]. Opioid consumption was significantly lower in the ESPB group than in the control group (MD −4.72, 95% CI −6.00 to −3.44, *p* < 0.001) (Figure 3). A moderate level of heterogeneity was observed among the studies (I^2^ = 50%; *p* = 0.06). 

**Figure 4:**
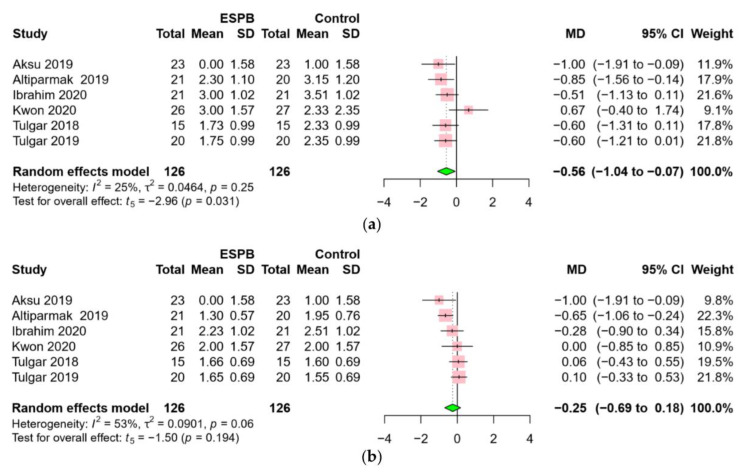
Forest plot for pain scores: (**a**) postoperative 12 h, (**b**) postoperative 24 h. ESPB provided lower pain scores until postoperative 12 h. Abbreviations: ESPB, erector spinae plane block; MD, mean difference; SD, standard deviation; CI, confidence interval.

Six RCTs [16,17,19,20,22,23], including 252 patients, reported pain severity using a visual analog scale (VAS) or numerical rating scale (NRS) of 0 to 10, at each predefined time point. This meta-analysis demonstrated a significant difference in the 12 h pain scores (MD −0.56, 95% CI −1.04 to −0.07, *p* = 0.031) (Figure 4a), whereas no significant difference in the 24 h pain scores between the two groups was observed (MD −0.25, 95% CI −0.69 to 0.18, *p* = 0.194) (Figure 4b). A low to moderate level of heterogeneity was found across the studies.

**Figure 5:**
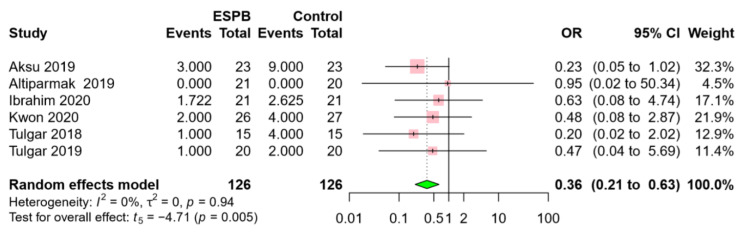
Forest plot for postoperative nausea and vomiting (PONV). ESPB reduced the incidence of PONV. Abbreviations: ESPB, erector spinae plane block; OR, odds ratio; CI, confidence interval.

The incidence of PONV was reported in 6 RCTs, including 252 patients [16,17,19,20,22,23]. The incidence of PONV was significantly lower in the ESPB group than in the control group (OR 0.36, 95% CI 0.21 to 0.63, *p* = 0.005) (Figure 5). A low level of heterogeneity was found among the studies (I^2^ = 0%; *p* = 0.94).

### 3.4. Erector Spinae Plane Block vs. Oblique Subcostal Transversus Abdominis Plane Block

**Figure 6:**
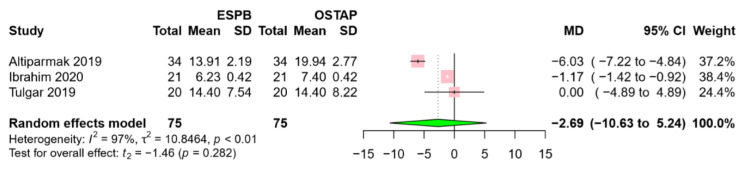
Forest plot for postoperative opioid consumption. Opioid consumption was comparable between the ESPB group and the OSTAP group. Abbreviations: ESPB, erector spinae plane block; OSTAP, oblique subcostal transversus abdominis plane block; SD, standard deviation; MD, mean difference; CI, confidence interval.

Three RCTs [18,19,23], including 75 patients in the ESPB group and the OSTAP group, reported postoperative opioid consumption. There was no significant difference in 24 h opioid consumption between the two groups (MD −2.96, 95% CI −10.63 to 5.24, *p* = 0.282) (Figure 6). A high level of heterogeneity was observed among the studies (I^2^ = 97%; *p* < 0.01).

**Figure 7:**
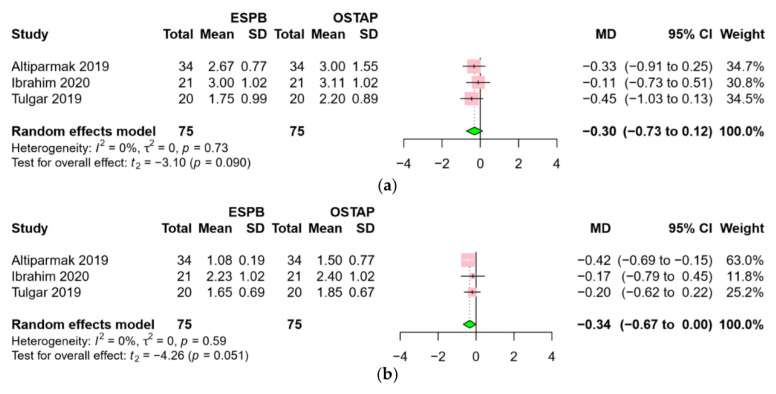
Forest plot for pain scores: (**a**) postoperative 12 h, (**b**) postoperative 24 h. Postoperative pain scores were comparable between the ESPB group and the OSTAP group. Abbreviations: ESPB, erector spinae plane block; OSTAP, oblique subcostal transversus abdominis plane block; MD, mean difference; SD, standard deviation; CI, confidence interval.

Three RCTs [18,19,23], comparing the ESPB group with the OSTAP group, reported data for postoperative pain scores at 12 h and 24 h. Pain scores were comparable between the two groups at both postoperative 12 h (MD −0.30, 95% CI −0.73 to 0.12, *p* = 0.090) (Figure 7a) and postoperative 24 h (MD −0.34, 95% CI −0.67 to 0.00, *p* = 0.051) (Figure 7b). A low level of heterogeneity was found across the RCTs at both time points (I^2^ = 0%).

**Figure 8:**
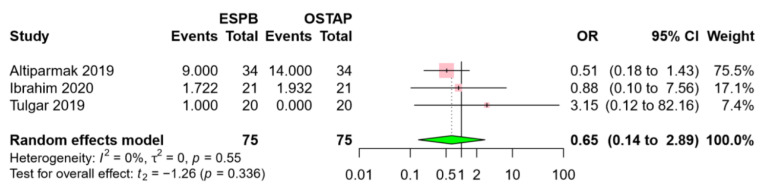
Forest plot for postoperative nausea and vomiting. The incidence of PONV was similar between the ESPB group and the OSTAP group. Abbreviations: ESPB, erector spinae plane block; OSTAP, oblique subcostal transversus abdominis plane block; OR, odds ratio; CI, confidence interval.

Three RCTs also reported the incidence of PONV [18,19,23]. The incidence of PONV was comparable between the two groups (OR 0.65, 95% CI 0.14 to 2.89, *p* = 0.336) (Figure 8). A low level of heterogeneity existed across the RCTs (I^2^ = 0%, *p* = 0.55).

## 4. Discussion

The results of this meta-analysis showed that ESPB reduced postoperative opioid consumption, pain scores, and the incidence of PONV. This is the first meta-analysis to demonstrate the analgesic efficacy of ESPB in patients undergoing LC. However, ESPB could not provide better analgesia compared to OSTAP block in patients undergoing LC.

The important finding of the current report is that ESPB reduced postoperative opioid consumption. This concurs well with the results of previous meta-analyses [6,7,26]. Opioid has long been used as a means to manage acute postoperative and postprocedural pain; however, a recent study reviewing clinical and administrative data from 135,379 adult patients receiving opioids after hospital-based surgeries or endoscopic procedures reported that 10.6% of the patients experienced opioid-related adverse events, which were related to poor outcomes, including increased inpatient mortality, prolonged length of hospital stay, and higher 30-day readmission rates [27]. In addition, given the current opioid crisis and its related morbidity and mortality, it is more important than ever to manage postoperative pain while minimizing the use of strong opioids [28]. To date, multimodal analgesia has been the standard of care for postoperative pain control to reduce opioid-related adverse effects [28]. In the aspect of multimodal analgesic technique, the demonstration of the efficacy of ESPB in reducing postoperative opioid consumption in this analysis might help to achieve such opioid-sparing analgesia and might be one of the valuable efforts to deal with the risk of the opioid crisis. 

The present analysis also showed that ESPB is associated with a reduction of pain scores until postoperative 12 h. Appropriate pain management is obviously an important aspect of perioperative anesthetic and surgical care. Additionally, acute surgical pain is a significant risk factor for the development of chronic pain and thereby a key target for intervention in reducing the risk of chronic postsurgical pain (CPSP) [29]. Furthermore, a previous study demonstrated that early visceral pain was associated with chronic pain development in patients undergoing LC [30]. To decrease the incidence of chronic pain development, the use of aggressive multimodal treatment methods that combine regional anesthesia, analgesia, and other analgesic medications is recommended during the perioperative period [29]. ESPB represents a promising option for perioperative analgesia and may play an important role in reducing CPSP after LC.

PONV is one of the most common and distressing complications after anesthesia and surgery. The general incidence of PONV can be as high as 80% in a subset of high-risk patients [31]. Perioperative use of opioid increases the risk of PONV in a dose-dependent manner [31], and ESPB reduced the incidence of PONV, which could be due to the reduction of postoperative opioid consumption in the current study.

Contrarily, this meta-analysis found no significant differences in postoperative opioid consumption, pain scores, and the incidence of PONV between the ESPB group and the OSTAP group. Given that these findings are based on a limited number of RCTs, the results from such analyses should consequently be treated with caution. Further studies on the comparison between ESPB and OSTAP block are therefore suggested in order to establish appropriate regional analgesia for patients undergoing LC. 

The result of this study showed low to moderate level of heterogeneity except for postoperative opioid consumption between the ESPB group and the OSTAP group. Although positive aspects of ESPB in terms of postoperative analgesia were described in the present study, heterogeneity remained to be answered since a moderate to high level of heterogeneity was found in postoperative opioid consumption. This could be explained by several factors, including different types of local anesthetics (bupivacaine, ropivacaine, or lidocaine), various concentrations of local anesthetics (0.25–0.5%), and different targets of the spinous process (T7, T8, or T9). An amount of 20 mL of 0.25% bupivacaine was used in most studies [16,18,19,21], followed by 20 mL of 0.375% bupivacaine in two studies [17,22]. In addition, pain control protocols after surgery were slightly varied among the studies (Table S3). Furthermore, two studies performed additional regional block for postoperative analgesia. Bupivacaine was infiltrated into trocar sites in the control group [19], or ropivacaine was administered between the rectus sheath and the rectus abdominis muscle in all groups [20].

This study has a few limitations. First, dermatomal sensory testing of the block was not performed in all RCTs. Thus, the success or failure rate of ESPB remains unknown, and this might have influenced the results of our analyses. Second, although we converted the doses of various types of opioid to morphine-equivalent doses, we cannot completely rule out the effect of different types of opioid on our results. Third, the result of this study showed that the analgesic effect of ESPB is limited to 12 h postoperatively (acute postoperative pain). However, a more prolonged analgesic effect of ESPB should be obtained to prove its impact on the prevention of chronic postsurgical pain. Further studies with more prolonged follow-up are needed to establish the effect of ESPB on chronic postoperative pain or postoperative clinical syndrome. 

## 5. Conclusions

In conclusion, the present study showed that ESPB reduced postoperative opioid consumption, pain scores, and the incidence of PONV in patients undergoing LC. Further investigations are needed for ESPB to be routinely implemented as appropriate regional analgesia for patients undergoing LC.

## Figures and Tables

**Table 1:** Baseline characteristics of the included randomized controlled trials (RCTs) (n = 8).

Study	Sample Size			Local Anesthetics	Level	PCA Regimen
	ESPB	Control	OSTAP			Bolus–Infusion–Lockout
Aksu 2019 [16]	23	23		0.25% BUPI 20 mL	T8	MP, 1–6 mg/hr–8 min
Altiparmak 2019 (1) [17]	21	20		0.375% BUPI 20 mL × 2	T7	TMD, 10 mg–0–20 min
Altiparmak 2019 (2) [18]	34		34	0.25% BUPI 20 mL × 2	T7	TMD, 20 mg–0–15 min
Ibrahim 2020 [19]	21	21	21	0.25% BUPI 20 mL × 2	T8	MP, 1 mg–0–12 min
Kwon 2020 [20]	26	27		0.20% ROPI 20 mL × 2	T7	-
Peker 2020 [21]	39	42		0.25% BUPI 20 mL × 2	T7	-
Tulgar 2018 [22]	15	15		0.375% BUPI 20 mL × 2	T9	TMD, 10 mg–0–20 min
Tulgar 2019 [23]	20	20	20	0.5% BUPI 20 mL + 2% LDC 10 mL+ NS 10 mL	T9	TMD, 10 mg–0–20 min

ESPB, erector spinae plane block; OSTAP, oblique subcostal transversus abdominis plane block; BUPI, bupivacaine; ROPI, ropivacaine; LDC, lidocaine; NS, normal saline; PCA, patient-controlled analgesia; MP, morphine; TMD, tramadol.

## Supplementary Materials

The following are available online at https://www.mdpi.com/2077-0383/9/9/2928/s1, Table S1: Search strategy for each database; Table S2: Details for judgement for each risk of bias for randomized controlled trials; Table S3: Standard analgesia protocols for each study.
