# Supplementary material for: The Effects of Erector Spinae Plane Block in Terms of Postoperative Analgesia in Patients Undergoing Laparoscopic Cholecystectomy: A Meta-Analysis of Randomized Controlled Trials
Source: J Clin Med. 2020 Sep 10;9(9):2928. doi: 10.3390/jcm9092928 (PMC7564953; doi:10.3390/jcm9092928)
Supplement: Supplementary file 1 [file jcm-09-02928-s001.pdf]

Table S1. Search strategy for each database.

Database	Order	Keywords	Results
PubMed	#1	"erector spinae plane"[TIAB] or "erector spinae plane block"[TIAB] or “erector spinae block”[TIAB] or ESB[TIAB] or ESP[TIAB] or ESPB[TIAB]	4272
	#2	#1 AND HSSS(S)	554
EMBASE	#1	‘erector spinae plane’:ab,ti or ‘erector spinae plane block’:ab,ti or ‘erector spinae block’:ab,ti or ESB:ab,ti or ESP:ab,ti or ESPB:ab,ti	6109
	#2	‘crossover procedure'/exp OR 'crossover procedure' OR 'double blind procedure'/exp OR 'double blind procedure' OR 'randomized controlled trial'/exp OR 'randomized controlled trial' OR 'single blind procedure'/exp OR 'single blind procedure' OR random* OR factorial* OR crossover* OR 'cross over' OR 'cross-over' OR placebo* OR (doubl* AND blind*) OR (singl* AND blind*) OR assign* OR allocat* OR volunteer*	2605829
	#3	#9 AND #10	555
	#4	#3 AND [embase]/lim	454
CENTRAL	#1	"erector spinae plane":ti,ab,kw or "erector spinae plane block":ti,ab,kw or “erector spinae block”:ti,ab,kw or ESB:ti,ab,kw or ESP:ti,ab,kw or ESPB:ti,ab,kw	653
	#2	#1 in Trials	651
CINAHL	S1	TI(erector AND spinae AND plane) OR AB(erector AND spinae AND plane)	38
	S2	TI(ESB OR ESP OR ESPB) OR AB(ESB OR ESP OR ESPB)	172
	S3	S1 OR S2	189
Web of Science	#1	TS=(erector spinae plane block) OR TS=(erector spinae block)	98

Table S2. Details for judgement for each risk of bias for randomized controlled studies.

Study	Bias	Author's judgement	Reason for judgement
Aksu 2019	Random sequence generation (selection bias)	Low	Randomization was performed according to computer-generated random number tables,
	Allocation concealment (selection bias)	Low	Allocation to treatment group was done using the sealed opaque envelope technique.
	Blinding (performance bias)	Unclear	The control group did not receive sham injection but ESP technique was performed under sedation.
	Blinding (detection bias)	Low	A pain nurse blinded to the procedure did all data collection.
	Incomplete outcome data (attrition bias)	Low	All patients completed the study and there were no losses.
	Selective reporting (reporting bias)	Low	All pre-specified and expected outcomes are reported.
	Other bias	Low	No other bias was detected.
Altiparmak 2019 (1)	Random sequence generation (selection bias)	Low	The enrolled patients were randomly allocated into two groups, based on a computerized randomization table created by a researcher who was not involved in the study.
	Allocation concealment (selection bias)	Low	Sealed envelope
	Blinding	Low	In the second group (control), patients received the same bilateral block with 40 ml of isotonic saline

	(performance bias)		solution to minimize the placebo effect.
	Blinding (detection bias)	Low	The patients were evaluated with NRS of 11 points by another anesthesiologist, in a blind character for the study groups.
	Incomplete outcome data (attrition bias)	Low	Some patients were excluded from the final analysis in both groups, but reasons for this are both reported and balanced across groups.
	Selective reporting (reporting bias)	Low	All pre-specified and expected outcomes are reported.
	Other bias	Low	No other bias was detected.
Altiparmak 2019 (2)	Random sequence generation (selection bias)	Low	Patients were randomly allocated into two groups based on a computerized randomization table created by a researcher who was not involved in the study.
	Allocation concealment (selection bias)	Low	For each randomized patient, the OR anesthesiologist took the corresponding sealed envelope from a folder, which indicated the treatment to be assigned to the patient.
	Blinding (performance bias)	High	Single blinded study
	Blinding (detection bias)	Low	The NRS scores were recorded by an anesthesiologist who was blinded to the group allocation.
	Incomplete outcome data (attrition bias)	Low	Some patients were excluded from the final analysis in both groups, but reasons for this are both reported and balanced across groups.
	Selective reporting	Low	All pre-specified and expected outcomes are

	(reporting bias)		reported.
	Other bias	Low	No other bias was detected.
Ibrahim 2020	Random sequence generation (selection bias)	Low	We utilized the sealed envelope method and computer-generated random numbers and then kept with a pharmacist.
	Allocation concealment (selection bias)	Low	The original random allocation sequences were kept in a secure place and a copy was used instead.
	Blinding (performance bias)	Low	ESP and OSTAP with isotonic saline for blinding
	Blinding (detection bias)	Low	Both participants and anesthetist responsible for data collection were blinded to the treatment group.
	Incomplete outcome data (attrition bias)	Low	All patients completed the study and there were no losses.
	Selective reporting (reporting bias)	Low	All pre-specified and expected outcomes are reported.
	Other bias	Low	No other bias was detected.
Kwon 2020	Random sequence generation (selection bias)	Low	Patients were randomized into two groups according to a computer-generated randomization schedule
	Allocation concealment (selection bias)	Unclear	Not described
	Blinding (performance bias)	High	Single blinded study
	Blinding (detection bias)	Low	Data collection was performed by the blinded study research coordinator or a blinded study investigator.

	Incomplete outcome data (attrition bias)	Low	Some patients were excluded from the final analysis in both groups, but reasons for this are both reported and balanced across groups.
	Selective reporting (reporting bias)	Low	All pre-specified and expected outcomes are reported.
	Other bias	Low	No other bias was detected.
Peker 2020	Random sequence generation (selection bias)	Low	Patients were randomly divided into two groups whether the ESP block was performed or not by a computer program
	Allocation concealment (selection bias)	Unclear	Not described
	Blinding (performance bias)	Unclear	The control group did not receive sham injection but ESPB group received the block under sedation.
	Blinding (detection bias)	Unclear	Not described
	Incomplete outcome data (attrition bias)	Unclear	No flow diagram
	Selective reporting (reporting bias)	Low	All pre-specified and expected outcomes are reported.
	Other bias	Low	No other bias was detected.
Tulgar 2018	Random sequence generation (selection bias)	Low	Upon ward admission, a random ID was assigned to each patient. Simple randomization in the operating room was performed using the closed envelope method to determine which group the patient would be included in.
	Allocation concealment (selection bias)	Low	
	Blinding	Unclear	The control group did not receive sham injection

	(performance bias)		but all blocks were performed under sedoanalgesia.
	Blinding (detection bias)	Low	The random ID assigned to each patient was used when collecting all patient data in the ward postoperatively. This data was therefore collected blindly.
	Incomplete outcome data (attrition bias)	Low	One patient in each group was excluded from the final analysis in both groups, but reasons for this are both reported and balanced across groups.
	Selective reporting (reporting bias)	Low	All pre-specified and expected outcomes are reported.
	Other bias	Low	No other bias was detected.
Tulgar 2019	Random sequence generation (selection bias)	Low	The sealed envelope technique was used to randomize patients into groups.
	Allocation concealment (selection bias)	Low	Same as above.
	Blinding (performance bias)	High	Single blinded study
	Blinding (detection bias)	Low	Recovery room and ward follow-up was performed by medical staff who were blinded to which group the patient was in.
	Incomplete outcome data (attrition bias)	Low	All patients completed the study and there were no losses.
	Selective reporting (reporting bias)	Low	All pre-specified and expected outcomes are reported.
	Other bias	Low	No other bias was detected.

Table S3. Details for judgement for each risk of bias for randomized controlled studies.

Study	At the end of surgery	Rescue analgesic	Routine analgesia
Aksu 2019	Paracetamol 1g + TMD 100 mg		MP PCA
Altiparmak 2019 (1)	75 mg dexketoprofen	MP 4mg (NRS≥4)	TMD PCA
Altiparmak 2019 (2)		MP 4mg (NRS≥4)	TMD PCA
Ibrahim 2020		FTN 15-20 mcg or MP 1-2 mg or pethidine 15-30 mg	Paracetamol 1g q 6h + MP PCA
Kwon 2020		PACU: FTN 0.4mcg/kg (NRS≥4)	50 mg dexketoprofen + 50 mg TMD or 25mg meperidine
Peker 2020		1 mg/kg TMD (NRS≥4)	
Tulgar 2018	Paracetamol 1g + tenoxicam 20 mg	FTN 25 mcg (NRS≥4)	Paracetamol 1g q 8h + TMD PCA
Tulgar 2019	Paracetamol 1g + tenoxicam 20 mg	FTN 25 mcg (NRS≥3)	Paracetamol 1g q 8h + TMD PCA

MP = morphine, TMD = tramadol, FTN = fentanyl, PCA = patient-controlled analgesia
